# Epithelial-mesenchymal transition and sunitinib resistance in renal cell carcinoma: mechanisms and therapeutic strategies

**DOI:** 10.3389/fphar.2026.1761280

**Published:** 2026-02-26

**Authors:** Mingkai Zhang, Yirui Zhang, Fan Shen, Maoli Yan, Pengfei Cheng, Jing Teng, Mengqin Zou, Wendi Yao, Zhifeng Wang, Wen Li

**Affiliations:** 1 CQMU - University of Leicester Joint Institute, Chongqing Medical University, Chongqing, China; 2 Center for Medical Epigenetics, School of Basic Medical Sciences, Chongqing Medical University, Chongqing, China; 3 Department of Urology, Henan Provincial People’s Hospital, Zhengzhou University People’s Hospital, Henan University People’s Hospital, Zhengzhou, Henan, China

**Keywords:** drug resistance, epithelial-mesenchymal transition, renal cell carcinoma, sunitinib, therapeutic strategies

## Abstract

Renal cell carcinoma (RCC) is a prevalent, highly aggressive malignant tumor that affects the urinary system. RCC has a pronounced propensity for metastasis. Despite the widespread use of sunitinib as first-line therapy for advanced RCC, the occurrence of primary and acquired resistance is frequent and presents significant challenges for effective clinical management. Epithelial–mesenchymal transition (EMT) induction is mediated by hypoxia-HIF signaling, chronic inflammatory stimulation, stromal-tumor cell interactions, and metabolic reprogramming, which confers increased cellular plasticity, migratory potential, and survival benefits. EMT activation is closely associated with reorganization of cellular signaling networks under tumor microenvironment stress, the initiation of alternative angiogenic pathways, and the enhanced anti-apoptotic capacity, all of which contribute to the development of sunitinib resistance. This review systematically summarizes current evidence involving the molecular basis of EMT-driven sunitinib resistance in RCC and investigates potential therapeutic targets, establishing a conceptual foundation for the development of novel strategies to counteract resistance and enhance clinical efficacy.

## 1 Introduction

According to GLOBOCAN 2022 statistics, there were approximately 434,419 new cases and 155,702 deaths of kidney cancer worldwide in 2022 (Bray et al., 2024). Renal cell carcinoma (RCC) arises from the epithelial cells of the renal tubules and accounts for >90% of renal tumors (Hsieh et al., 2017). Patients with RCC are often asymptomatic in the early stage, whereas the classical triad (haematuria, pain, and a palpable abdominal mass) is uncommon and typically reflects an advanced stage (Vasudev et al., 2020). Approximately 30% of patients with RCC present with metastatic disease at the time of diagnosis (Capitanio et al., 2019). The prognosis for metastatic RCC is poor with a median survival of approximately 13 months and a 5-year survival rate <10% (Cairns, 2011). Therefore, selecting an appropriate therapeutic strategy is crucial for improving patient outcomes and reducing mortality.

Current RCC management includes nephrectomy, targeted therapy, immunotherapy, cytokine therapy, and radiotherapy (Bahadoram et al., 2022). Among the treatment options, approximately 30% of patients have a post-nephrectomy recurrence. Radiotherapy has historically been regarded as ineffective for RCC due to inherent radio-resistance However, radiotherapy is generally utilized for palliative care and management of bone and brain metastases (Makhov et al., 2018; Uroweb, 2026). Cytokine therapy, including interleukin-2 and interferon-alpha, is associated with considerable toxicity and limited efficiency (Jonasch et al., 2014). Immunotherapy holds significant potential but has limited clinical efficacy based on heterogeneous patient responses and immune-related adverse events (Drobner et al., 2023). In this context, targeted therapy, particularly tyrosine kinase inhibitors (TKIs) such as sunitinib, has become a cornerstone in the management of advanced RCC by inhibiting angiogenesis and tumor cell proliferation. Nevertheless, therapeutic efficacy is frequently compromised by acquired resistance, intratumoral heterogeneity, and immune evasion. Acquired resistance represents the principal limitation, ultimately leading to treatment failure and cancer progression (Azijli et al., 2015).

An emerging mechanism underlying drug resistance in RCC is the epithelial-mesenchymal transition (EMT), a dynamic biological process in which epithelial cells acquire mesenchymal traits. This phenotypic transition enhances cellular invasiveness, metastatic potential, and survival capacity, thereby contributing to sunitinib resistance (Sweeney et al., 2023). Other factors include activation of pro-survival signaling pathways, overexpression of drug efflux transporters, and metabolic reprogramming (Wang et al., 2023). This review aims to explore the role of EMT in facilitating sunitinib resistance in RCC and offer insights that may guide future basic research and inform clinical strategies.

## 2 Sunitinib in RCC: molecular mechanisms and clinical applications

Although sunitinib remains the standard first-line therapy for advanced and metastatic RCC, the therapeutic outcomes among patients are highly heterogeneous. This variability is mostly driven by genetic, metabolic, and microenvironment factors that influence drug efficacy and toxicity. Therefore, this section provides an overview of sunitinib pharmacologic properties, major clinical findings, and the therapeutic challenges that shape the role of sunitinib in RCC management.

### 2.1 Molecular mechanisms of Sunitinib’s anti-tumor activity in RCC

**FIGURE 1:**
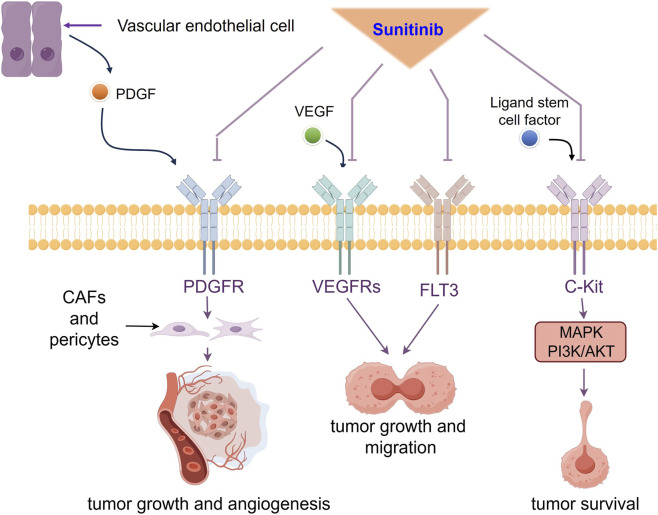
The main receptor tyrosine kinases targeting by sunitinib in RCC. Sunitinib concurrently blocks PDGFR, VEGFRs, FLT3 and c-KIT. PDGFR inhibition disrupts pericyte/CAF-derived stromal support and angiogenic maturation. VEGFRs and FLT3 suppression limits cell proliferation and dissemination. The blockade of c-KIT downregulates MAPK and PI3K/AKT signaling.

Sunitinib is classified as a multi-targeted TKI that inhibits angiogenesis and tumor growth by targeting tyrosine kinase (RTK) receptors, including vascular endothelial growth factor receptors (VEGFRs), platelet-derived growth factor receptors (PDGFRs), cellular homolog of the v-kit Hardy-Zuckerman 4 feline sarcoma viral oncogene (c-Kit/kit), and FMS-like tyrosine kinase 3 (FLT3) (Sweeney et al., 2023) (Figure 1).

Vascular endothelial growth factor (VEGF) mediates hypoxia-induced neovascularization and increases vascular permeability to regulate angiogenesis (Itatani et al., 2018). Sunitinib has been reported to obstruct the interaction between VEGF and VEGFR, which in turn inhibits angiogenesis and restricts proliferation and metastasis (Kamli et al., 2019). Platelet-derived growth factor (PDGF) is synthesized by vascular endothelial cells to modulate the activities of pericytes and fibroblasts through interaction with PDGFR-α and PDGFR-β (Strell et al., 2024). Sunitinib has been shown to diminish stromal support within the tumor microenvironment (TME) by inhibiting receptor activities, thereby suppressing tumor growth and angiogenesis (Itatani et al., 2018). C-Kit activates the mitogen-activated protein kinase (MAPK) and the phosphatidylinositol 3'-kinase (PI3K/AKT) through an interaction with the c-Kit ligand and facilitates the proliferation, invasion, and angiogenesis of RCC (Sheikh et al., 2022). Sunitinib blocks transduction of survival signals by competitively binding to receptors, thereby inhibiting tumor progression (Marech et al., 2014). FLT3 is frequently overexpressed in a range of hematologic and solid tumors, activating mutations that result in constitutive kinase signaling are commonly observed (Meshinchi and Appelbaum, 2009). As a multi-targe TKI, sunitinib inhibits FLT3 autophosphorylation in a dose-dependent manner, leading to reduced proliferation and migration in human meningioma cell lines (Andrae et al., 2012).

### 2.2 Clinical utility and therapeutic landscape of sunitinib in RCC

Sunitinib is a standard first-line therapy for advanced and metastatic RCC, particularly clear cell type (ccRCC) (Ansari et al., 2010; Motzer et al., 2006). A phase III trial involving metastatic ccRCC demonstrated superior efficacy of sunitinib compared to interferon-αwith a median progression-free survival (PFS) of approximately 11 months and higher response rates (Motzer et al., 2007). A recent 5-year follow-up evaluation of patients with advanced RCC showed a median overall survival (OS) approaching 3 years (Rini et al., 2025). An expanded access study reported a PFS of approximately 11 months and an OS of approximately 18 months with clinical benefits noted in patients with brain metastases, poor performance, and even non-ccRCC (Gore et al., 2009). Common toxicities of sunitinib include fatigue, nausea, hypertension, and hand-foot syndrome, with require dose adjustments or hospitalization (Ansari et al., 2010). Despite initial efficacy, sunitinib is rarely curative owing to the inevitable development of resistance (Le Tourneau et al., 2007). Clinically, approximately 30% of patients with RCC exhibit intrinsic resistance, while most patients acquire resistance within 6–15 months of treatment (Sharma et al., 2021). The clinically confirmed resistance mechanisms in RCC are summarised in Table 1, providing a framework for the following section. Given the central role of EMT in mediating drug evasion and crosstalk with other resistance pathways, elucidating biological function is critical for improving therapeutic outcomes in RCC.

**TABLE 1:** Confirmed sunitinib resistance mechanisms in RCC patients.

Drug resistance	Mechanism	Ref.
Angiogenesis pathway activation	Upregulated VEGF/PDGFR signaling drives angiogenesis and supports resistance	Zucca et al. (2018)
Hypoxia and HIF signaling activation	Hypoxia activates HIF signaling, enhancing angiogenesis and tumor adaptation	Wierzbicki et al. (2019), Garcia-Donas et al. (2013)
Drug efflux mechanisms	ABC transporter overexpression increases efflux and lowers intracellular drug levels	Wang et al. (2023)
Lysosomal sequestration	Sunitinib is sequestered in lysosomes, preventing it from reaching its targets	Azijli et al. (2015), Gotink et al. (2011), Giuliano et al. (2015)
Abnormal drug uptake and efflux systems	Altered transporters impair drug uptake and retention in tumor cells	(Sun et al., 2021; Springer Nature, 2026g)
Drug exposure concentration variability	Pharmacokinetic variability reduces systemic exposure and causes resistance	(Pharmacodynamic meta analysis, 2026; Westerdijk et al., 2021; Springer Nature, 2026h; Henriksen et al., 2024)

## 3 Biological characteristics and functional roles of EMT in RCC

RCC cells undergo cytoskeletal reorganization, extracellular matrix (ECM) remodeling, and modulation of intercellular adhesion that is mediated by transcription factor regulation and supported by tumour-associated immune cells, enabling RCC cells to endure therapeutic stress and thereby facilitate invasion, metastasis, and colonization. Accordingly, this section presents an overview of the molecular features and functional implications of EMT in RCC with a particular focus on the regulatory networks that govern tumor aggressiveness and contribute to the emergence of drug resistance.

### 3.1 EMT: molecular hallmarks and roles in metastasis

**FIGURE 2:**
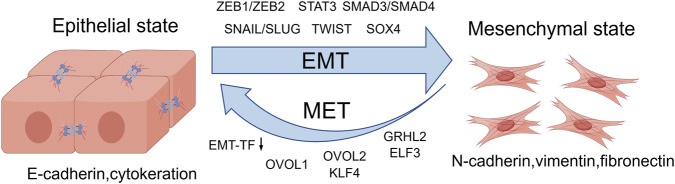
EMT involves the loss of epithelial markers and the acquisition of mesenchymal traits, driven by EMT-TFs STAT, SMAD4, and SOX4, whereas MET is characterized by reduced EMT-TF activity and reactivation of epithelial-promoting factors OVOL1/2, GRHL2, KLF4, and ELF3.

EMT is marked by the conversion of epithelial cells into a mesenchymal phenotype, which involves alterations in cell adhesion molecules and cytoskeletal structures and shifts towards enhanced invasiveness and migratory capacity (Pastushenko and Blanpain, 2019). This transition is primarily characterized by downregulation of epithelial markers, such as E-cadherin and cytokeratin, and upregulation of mesenchymal markers, including N-cadherin, vimentin, and fibronectin (Huang et al., 2022). E-cadherin is replaced by N-cadherin throughout the EMT, resulting in reduced cell-cell adhesion. This alteration promotes the conversion of epithelial cells from a stationary, sheet-like configuration to motile and individual entities, thereby driving a substantial shift in cellular identity (He and Magi-Galluzzi, 2014; Kaufhold and Bonavida, 2014). EMT is regulated by a core set of transcription factors at the molecular level, including Snail (*Snal1*), Slug (*Snal2*), basic helix-loop-helix factors (TWIST1) and the zinc finger E-box-binding homeobox proteins (ZEB1 and ZEB2) (Zeisberg and Neilson, 2009). These factors are known as “Epithelial-mesenchymal transition transcription factors (EMT-TFs)”, and function in initiating mesenchymal gene expression and endowing cells with migratory and invasive capabilities (Brabletz et al., 2021; Boutet et al., 2006) In addition, beyond EMT-TFs, STAT3, SMAD3/4, and SOX4 also play important roles in driving the EMT program and promoting metastatic progression (Nature Communications, 2026; Nature Cell Biology, 2026a; Cancer Research, 2026a) (Figure 2).

EMT prepares tumor cells for invasion and metastasis by inducing cell junction disassembly, cytoskeleton reorganization, and matrix metalloproteinases (MMPs) upregulation (Lu and Kang, 2019; Nagai et al., 2020). Cells that have undergone EMT intravasation into the bloodstream or lymphatic system as circulating tumor cells (CTCs). Although the formation of platelet cloaks around CTCs improves CTC survival, the overall metastatic efficiency remains extremely low (Quail et al., 2013; Singh et al., 2014; Luzzi et al., 1998; Gay and Felding-Habermann, 2011). Upon arrival at distant organs, CTCs are arrested and adhere to the vascular endothelium, subsequently traversing the endothelial layer and the ECM to infiltrate the parenchyma of the target organ (de Visser and Joyce, 2023; Gerstberger et al., 2023). This infiltration is often accompanied by mesenchymal-epithelial transition (MET), a reverse process that facilitates epithelial re-differentiation and supports metastatic colonization (Jolly et al., 2017). Conversely, metastatic colonization-associated MET is often accompanied by an overall decline of EMT-TFs, and can be promoted by epithelial-maintaining factors such as OVOL1/2, GRHL2, KLF4 and ELF3 (PLOS One, 2026; Cancer Biology Therapy, 2026; MDPI, 2026; Oncotarget, 2026) (Figure 2). Tumor cells gain the phenotypic plasticity necessary for effective colonization at distant sites through this dynamic transformation. Taken together, these processes highlight the pivotal function of EMT in facilitating tumor cells to complete the metastatic cascade.

### 3.2 Distinctive features of EMT in RCC

The induction and maintenance of EMT rely on multiple signaling pathways. Transforming growth factor-beta (TGF-β) is intimately involved in cellular functions, including proliferation, differentiation, adhesion, and migration, and is recognized as a key regulator of EMT initiation (Massagué, 2012; David and Massagué, 2018). TGF-β phosphorylates Smad2 and Smad3, which subsequently form a trimer with Smad4. This complex binds to DNA and promotes EMT-TFs expression, leading to downregulation of epithelial markers and upregulation of mesenchymal markers (Hao et al., 2019; Xu et al., 2009). The phosphoinositide 3-kinase (PI3K)-AKT-mTOR and Wnt/β-catenin pathways have been shown to regulate transcriptional networks that govern cell migration, thereby contributing to EMT progression (Lamouille et al., 2012; Xue et al., 2024).

Unlike other solid tumors where EMT relies on TGF-β/Smad, PI3K–AKT, or Wnt/β-catenin signaling to drive cytoskeletal remodeling, EMT in RCC is predominantly driven by a kidney-specific hypoxic and metabolic microenvironment (Nature Reviews Molecular Cell Biology, 2026; Piva et al., 2016). A prototypical example is the VHL-HIF axis, which integrates hypoxia signaling with angiogenesis, metabolic reprogramming, and therapeutic resistance. This axis is pivotal in strengthening RCC cell survival, driving tumor growth, and amplifying metastatic potential (b). At the cellular level, RCC exhibits EMT-associated dedifferentiation features, most notably sarcomatoid transformation. This aggressive phenotype is defined by the downregulation of epithelial markers, concurrent upregulation of mesenchymal markers, and a marked increase in invasive potential (Čugura et al., 2024). Clinically, high expression of EMT-markers in RCC correlates with an unfavorable prognosis and reduced survival rates (Dumanskiy et al., 2013). While RCC retains the canonical EMT signaling architecture shared by other solid tumors, it exhibits a unique plasticity driven by the VHL-HIF-mediated hypoxia and metabolic ecosystem, underscoring the critical need to identify more RCC-tailored predictive biomarkers and actionable therapeutic targets.

### 3.3 Tumor microenvironment crosstalk in EMT

Recent single-cell sequencing studies indicated that high EMT-TF activity is associated with metastasis and reduced patient survival (Cancer Research, 2026b). Mesenchymal-like tumor cells are enriched in metastatic lesions, which are associated with poor survival outcomes (Cancer Research, 2026b). Similarly, two metastasis-associated gene expression programs were characterized by high expression of ECM components and EMT-related genes (Springer Nature, 2026a). Moreover, multi-region scRNA-seq atlases of ccRCC identified an EMT meta-program at invasive edges and single-cell analyses delineated metastasis-associated programs characterized by high ECM/EMT gene expression (Zvirblyte et al., 2024). An integrative mRNA analysis revealed that EMT-related genes, such as *IL-6*, are upregulated in sunitinib-resistant RCC, indicating roles in mediating tumor cell resistance (Deng et al., 2023).

**FIGURE 3:**
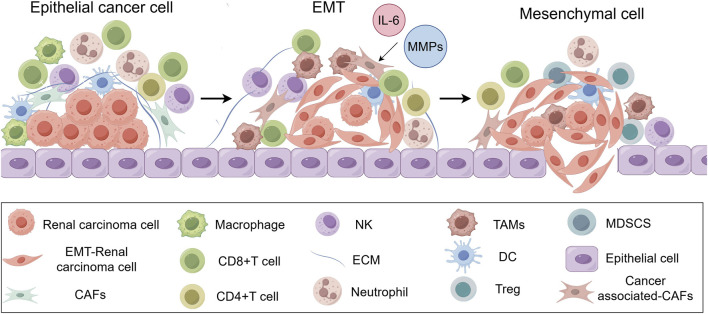
EMT and associated microenvironment alterations. The tumor microenvironment promotes EMT process through IL-6 and MMPs secretion and immune cell infiltration, reinforcing tumor progression and immune evasion.

In addition, the TME drives EMT by providing cytokine and ECM signals that promote a mesenchymal, invasive phenotype (Figure 3). Cancer-associated fibroblasts (CAFs) and tumor-associated macrophages (TAMs) within the TME secret factors including MMPs and interleukin-6 (IL-6), to induce EMT (Erin et al., 2020; Khan et al., 2023). ECM facilitates EMT initiation and tumor metastasis through remodeling the mechanical stiffness and degradation characteristics (Lu et al., 2012; Najafi et al., 2019). Mesenchymal-like cells exhibit elevated expression of immune checkpoint molecules, including PD-L1, PD-L2, and CTLA-4, to evade cytotoxic T lymphocyte (CTL)-mediated killing (Clinical Cancer Research, 2026a). Activation of EMT-TFs leads to immunosuppressive cells accumulation within the TME, including TAMs, myeloid-derived suppressor cells (MDSCs), and regulatory T cells (Tregs), thereby establishing an immunosuppressive microenvironment (Clinical Cancer Research, 2026a). Furthermore, EMT induces angiogenesis and metabolic reprogramming to support tumor cell survival (Xie et al., 2025). In summary, the crosstalk between a tumor and the TME establishes dynamic feedback that maintains the EMT phenotype and promotes cancer progression.

### 3.4 Translational relevance of EMT in RCC management

Although RCC originates from epithelial cells of the renal tubules, sarcomatoid features are frequently observed in advanced-stages (Mikami et al., 2015). Immunohistochemical and transcriptomic investigations revealed a marked downregulation of epithelial markers and an upregulation of mesenchymal markers in sarcomatoid (s)RCC (He and Magi-Galluzzi, 2014; Conant et al., 2011; Boström et al., 2012). Upregulation of EMT-TFs is positively correlated with higher TNM stages, Fuhrman nuclear grades, and metastatic potential (Mikami et al., 2015; Fang et al., 2013; Ohba et al., 2014). The *von Hippel-Lindau* (*VHL*) gene, a key tumor suppressor that is often inactivated in RCC, regulates the stability of hypoxia-inducible factor-1α (HIF-1α) (Zhang and Zhang, 2018). Loss of *VHL* leads to HIF-1α accumulation, which subsequently upregulates EMT-TFs to drive EMT and promote metastasis (Esteban et al., 2006; Pantuck et al., 2010; Schokrpur et al., 2016). In addition, *VHL*-deficient RCC secretes IL-6 to induce TAM polarization toward an M2 phenotype, which subsequently releases chemokine CCL18 and TGF-β to further accelerate EMT (Nguyen et al., 2022). The following section explores the connection between EMT activation and sunitinib resistance in RCC, highlighting the key challenges and discussing prospective approaches to overcome therapeutic resistance.

## 4 Mechanistic interplay between EMT and sunitinib resistance in RCC

EMT functions as a central mechanistic interface that enables RCC cells to circumvent the effects of sunitinib, converting epithelial plasticity into coordinated survival programs under therapeutic stress. This section summarizes EMT-associated resistance pathways, emphasizing the molecular crosstalk and clinical relevance to sunitinib resistance in RCC.

### 4.1 EMT-driven activation of pro-survival signaling pathways

**FIGURE 4:**
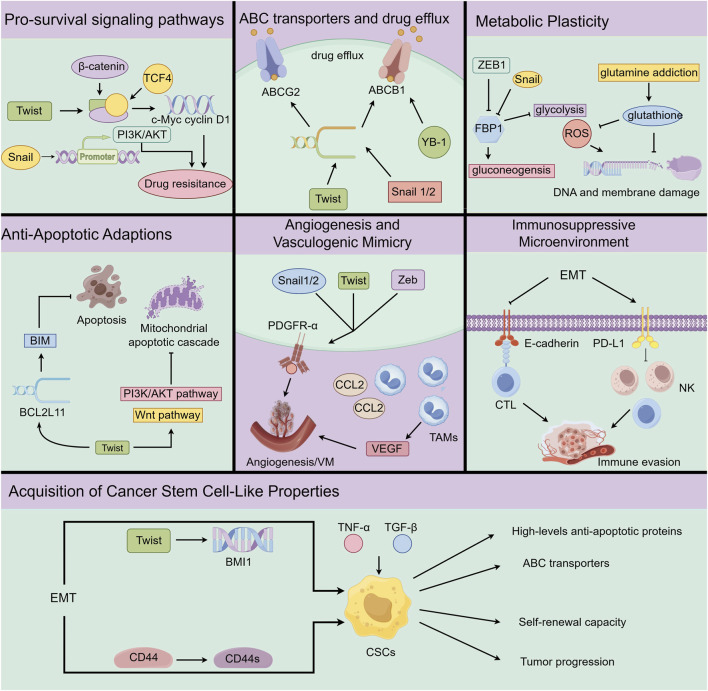
Mechanism of EMT-mediated sunitinib resistance in RCC. EMT activates pro-survival signaling (PI3K/AKT), enhances ABC transporter associated drug efflux, reprograms metabolism, confers TWIST-dependent apoptosis resistance, promotes TAM/VEGF-driven angiogenesis, alters surface proteins to evade immunity, and induces stem cell-like traits facilitating therapy escape.

EMT is closely associated with pro-survival signals activation. Among the EMT-associated resistance pathways, the PI3K-AKT is critical for the regulation of proliferation, survival, self-renewal, and resistance to apoptosis (Revathidevi and Munirajan, 2019). Hyperactivation of PI3K-AKT in RCC has been shown to diminish sunitinib sensitivity by upregulating HIF and promoting angiogenesis, thereby influencing therapeutic efficacy (Sun et al., 2023) (Figure 4). As a key EMT transcription factor, Snail has been shown to bind the AKT promoter region to enhance kinase activity and downstream signaling. This interaction further amplifies PI3K-AKT in mediating sunitinib resistance (Skrzypek et al., 2020; Oncogene, 2026a). EMT also converges on hypoxia-driven survival programs. The hypoxic TME maintains HIF-1α stability under anti-angiogenic therapy and activates β-catenin to promote RCC proliferation and drug resistance (Conley et al., 2012; Zhong et al., 2024). Similarly, TWIST has been shown to interact with β-catenin and TCF4, facilitating c-Myc and cyclin D1 expression. This activation contributed to the acquisition of stem-like features and promotes therapeutic resistance (Cai et al., 2024; Gao et al., 2022). In conclusion, EMT establishes signaling redundancy by activating pro-survival pathways, thereby diminishing the therapeutic efficacy of sunitinib and enabling cancer cells to evade treatment-mediated suppression.

### 4.2 Upregulation of ATP-binding cassette (ABC) transporters and drug efflux

Overexpression of ATP-binding cassette (ABC) transporters actively expels therapeutic agents from cancer cells (Zhong et al., 2024). P-glycoprotein (ABCB1) and breast cancer resistance protein (ABCG2) are two key transporters in RCC, the elevated expression of which has been significantly associated with poor 5-year survival and increased distant metastasis (Wang et al., 2017; Kadioglu et al., 2020) (Figure 3). These findings highlight the therapeutic significance of targeting ABC-mediated drug efflux, especially in the case of aggressive RCC. EMT-TFs have been shown to bind to ABC transporter promoter, resulting in upregulation of transcriptional activity (Du and Shim, 2016; Saxena et al., 2011). In addition, EMT activates Y-box binding protein (YB-1) to function as a crucial regulator of ABCB1 expression (D’Costa et al., 2020). Analysis of metastatic ccRCC (mccRCC) patient samples treated with sunitinib revealed elevated YB-1 and ABCB1, indicating a potential role of EMT in modulating drug efflux responses (D’Costa et al., 2020). Therefore, EMT-induced upregulation of these transporters significantly diminishes the intracellular bioavailability of sunitinib, thereby impairing therapeutic effectiveness (Tang et al., 2012). In summary, the EMT-driven upregulation of ABC transporter expression represents a pivotal mechanism underlying pharmacokinetic resistance, facilitating the active efflux of sunitinib in RCC and allowing the tumor to evade therapeutic intervention (Figure 4).

### 4.3 Metabolic reprogramming and plasticity in EMT-Transformed cells

Tumor cells frequently undergo metabolic reprogramming to adapt to external stress and sustain rapid proliferation, a process further amplified by EMT (Jia et al., 2021). EMT typically drives metabolic shift from oxidative phosphorylation toward aerobic glycolysis, a phenomenon commonly referred to as the “Warburg effect”, while simultaneously increasing dependence on glutamine, which is known as “glutamine addiction”, to meet elevated bioenergetic and redox demands (Sciacovelli and Frezza, 2017). Interestingly, Snail and ZEB1 epigenetically repress the key gluconeogenesis enzyme fructose-1,6-bisphosphatase (FBP1), promoting the metabolic transition towards glycolysis (Molecular and C ellular Biochemistry; Dong et al., 2013). The increased glycolysis leads to acidification of TME, which in turn facilitates angiogenesis and metastasis and confers a survival advantage to tumor cells under therapeutic stress (Beckert et al., 2006). Moreover, the dependence on glutamine observed in EMT-like tumor cells supports the synthesis of glutathione, a major antioxidant that counteracts reactive oxygen species (ROS) and safeguards cellular DNA and membranes against oxidative damage (Aboud et al., 2017). Resistant RCC cells exhibit increased glutamine uptake and glycolytic activity compared to sensitive cells (Sato et al., 2020). This effect is further associated with elevated antioxidant capacity and enhanced proliferation, which contribute to immune evasion and tumor progression (Amaro et al., 2024). In conclusion, EMT-driven metabolic reprogramming fulfils the energetic and survival requirements of tumor cells under treatment, thereby forming a critical foundation for therapeutic resistance (Figure 4).

### 4.4 EMT-associated anti-apoptotic adaptations

A hallmark of tumor cells exhibiting EMT is the pronounced resistance to apoptosis. This feature is frequently accompanied by upregulation of *Bcl-2* family genes and altered expression of anti-apoptosis factors (Ribatti et al., 2020; Inoue-Yamauchi and Oda, 2020). TWIST has been shown to bind to the promoter of the pro-apoptosis gene, *BCL2L11* (which encodes BIM), thereby repressing transcription and silencing a crucial trigger of programmed cell death (Zhang et al., 2007). In addition, TWIST not only elevates the Bcl-2/Bax ratio to favour anti-apoptosis dominance (Yochum et al., 2019) but also activates AKT and β-catenin to block the mitochondrial apoptotic cascade (Li and Zhou, 2011; Ma et al., 2019). Elevated Bcl-2 expression correlates with decreased BIM expression, which is strongly linked to decreased apoptosis (Mao et al., 2013; Hata et al., 2015). EMT-driven upregulation of anti-apoptotic capacity in RCC has been implicated in the development of sunitinib resistance (Figure 4). Sunitinib induces apoptosis through inhibition of STAT3 activity (Xin et al., 2009), which is diminished by enhanced anti-apoptosis capacity (Wu et al., 2022). A significant upregulation of Bcl-2 has been demonstrated with sunitinib-resistant RCC models (Kam et al., 2018). Therefore, even under sunitinib treatment, tumor cells may exploit EMT to activate anti-apoptotic pathways and suppress pro-apoptotic signaling to counteract the therapy-induced cytotoxic stress. In conclusion, EMT serves as a key mechanism that enables tumor cells to evade therapy-induced apoptosis, substantially contributing to the development of drug resistance.

### 4.5 EMT-induced angiogenesis and vasculogenic mimicry

RCC is characterized by a pronounced level of vascularization and sunitinib mediates the therapeutic effect by targeting angiogenesis to inhibit tumor growth (Astore et al., 2023). Nevertheless, EMT facilitates tumor cells to bypass conventional angiogenic pathways by adopting alternative vascularization strategies (PMC, 2026a). One such mechanism is vasculogenic mimicry (VM), a non-endothelial mode of neovascularization in which tumor cells generate vessel-like channels independent of endothelial cells, thereby sustaining nutrient supply even when classical angiogenesis is inhibited (He M. et al., 2022). EMT-TFs are significantly upregulated in tumor cells that exhibit VM structures (Liu et al., 2016), which are through to promote VM through sustaining stem-like characteristics and activating PDGFR-α (Tang et al., 2024; You et al., 2021). EMT further supports VM formation by recruiting stromal cells from the TME. Cancer cells that express *TWIST1* enhance *CCL2* transcription (Low-Marchelli et al., 2013), which facilitates TAM recruitment and promotes vascular remodelling (Lewis et al., 2000). TAMs adapt to hypoxic conditions induced by sunitinib in RCC, and further upregulate alternative angiogenic genes to support tumor survival (Santoni et al., 2013) (Figure 4). In summary, EMT-induced vascularization markedly diminishes therapeutic efficacy of sunitinib, representing a key mechanism by which tumors sustain survival under anti-angiogenic pressure.

### 4.6 EMT-sustained immunosuppressive microenvironment

EMT not only modifies the morphology and motility of tumor cells but also contributes to the formation of an immunosuppressive TME. Tumor cells typically undergo alterations in surface molecules during EMT and exhibit reduced infiltration by anti-tumor immune cells, thereby evading recognition and attack by CTLs and natural killers (NK) cells (Chae et al., 2018; PMC, 2026b). E-cadherin expressed on epithelial tumor cells can interact with integrin αEβ7 on CTLs, which facilitates their cytolytic activity (Franciszkiewicz et al., 2013). However, EMT-driven downregulation of E-cadherin significantly impairs CTL function and promotes immune escape (Floc’h et al., 2007). Moreover, programmed death ligand 1 (PD-L1) is markedly upregulated in sRCC and consequently suppresses immune-mediated tumor clearance (Joseph et al., 2015; World Journal of Urology, 2026). In addition, upregulation of *brachyury*, the T-box transcription factor and key EMT driver, has also been implicated in the induction of resistance to cytotoxicity mediated by CTL and NK cells (Hamilton et al., 2014; Hamilton et al., 2016). Sunitinib exerts immunomodulatory functions by inhibiting STAT3, thereby enhancing CTL and NK cells, while reducing the population of MDSCs and Tregs (Xin et al., 2009; Nature Medicine, 2026). In summary, the acquisition of an EMT phenotype enables RCC cells to evade immune-mediated destruction, preserving a pool of surviving cells that ultimately contributes to resistance against sunitinib treatment (Figure 4).

### 4.7 Acquisition of cancer stem cell-like properties via EMT

Cancer stem cells (CSCs) represent a distinct subpopulation of tumor cells characterized by a strong self-renewal capacity to initiate and sustain tumor growth (Olivares-Urbano et al., 2020). CSC biomarkers identified in RCC include CD44, CD133, CD105, ALDH1, and CXCR4 (Corrò and Moch, 2018; Lasorsa et al., 2023). CSCs are recognized as key contributors of tumor progression and therapeutic resistance (Mikami et al., 2015; NIM, 2026; Micucci et al., 2015; Xiao et al., 2017). The EMT-TFs also endow tumor cells with stem-like properties (Figure 4). TWIST binding to the regulatory region of BMI1 promotes cellular stemness (Nature Cell Biology, 2026b). ZEB1 represses the epithelial splicing regulator, ESRP1, leading to a shift in CD44 isoform expression towards the stem-associated variant. In turn, CD44 activates *ZEB1* transcription to sustain EMT and stemness (Preca et al., 2015). In addition, pro-inflammatory cytokines, including TNF-α and TGF-β, induce EMT and confer stem cell-like properties (Zhang et al., 2014; Singla et al., 2018). This interaction between EMT and CSCs facilitates phenotypic plasticity and promotes therapeutic evasion. CSCs present challenges to effective cancer therapy due to cellular dormancy, overexpression of anti-apoptotic surface proteins, metabolic reprogramming, and upregulation of ABC transporters (Steinbichler et al., 2018; Tanabe et al., 2020). Tumors in RCC with diminished stemness resulting from miR-381 overexpression demonstrate increased sensitivity to sunitinib (Lu et al., 2023). In summary, the EMT-CSC interplay enhances cell plasticity and supports the persistence of drug-resistant subpopulations, thereby presenting a major therapeutic challenge.

### 4.8 Sunitinib-induced EMT and tumor adaptive evolution

Notably, EMT is not solely a prerequisite for drug resistance but also serves as a downstream adaptive response to therapeutic pressure. Under the intense selective stress of chemotherapy, targeted therapy, or immunotherapy, residual tumor cells initiate a “Therapy-Induced EMT” program. By activating intracellular survival signaling networks, initially drug-sensitive epithelial tumor cells undergo dedifferentiation, transitioning into a highly plastic mesenchymal state. Accumulating evidence suggests that sunitinib exposure drives an EMT-like adaptive shift in RCC. In a chronic sunitinib-treatment model, RCC cells exhibit increased AXL and MET expression, which induces upregulation of EMT-related genes and is accompanied by enhanced migration and invasion (Oncogene, 2026b). VEGF-pathway blockade by sunitinib is shown to lead to accelerated metastatic dissemination, potentially driven by increased VEGFC-associated lymphatic routes (Cancer Cell, 2026; Dufies et al., 2017). Mechanistically, sunitinib-resistant RCC cells may also augment invasiveness by enhancing lysosome biogenesis and drug efflux (Springer Nature, 2026b). Sunitinib can promote VM to further consolidate plasticity-associated resistant phenotypes (He M. et al., 2022).

### 4.9 Co-evolution of EMT and drug resistance based on common signaling nodes

A “parallel regulation” between EMT and drug resistance is anchored in shared upstream signaling nodes. Multiple signaling pathways exhibit pleiotropic regulatory functions. Dysregulation of the Hippo-YAP simultaneously enhances RCC invasiveness and attenuate responsiveness to sunitinib, thereby coupling metastatic competence with therapeutic resistance (Fu et al., 2025). The Wnt/β-catenin pathway further illustrates this convergence: Wnt signaling integrates with other networks to drive EMT and promotes the development of sunitinib resistance through crosstalk with EMT-TFs (Cai et al., 2024). This intrinsic coupling ensures that tumor cells synchronously acquire therapeutic defiance alongside metastatic potential, thereby explaining the clinical observation that highly invasive tumors are frequently accompanied by a recalcitrant multidrug-resistant phenotype. Metabolic reprogramming, such as glutamine metabolism, not only augments tumor aggressiveness but also reshapes VEGFR-related signaling and resistance states (Morozumi et al., 2024). Furthermore, intercellular crosstalk within the TME, involving stromal and immune components, serves to attenuate the response to sunitinib and promote invasiveness, thereby substantiating the close linkage between metastatic progression and therapy resistance (Nagas et al., 2021).

## 5 Therapeutic strategies targeting EMT to overcome sunitinib resistance in RCC

Given the significance of EMT in driving sunitinib resistance, effects to target this process has emerged as a promising approach to restore treatment efficacy in RCC. Strategies to overcome EMT have been increasingly explored, including the modulation of EMT-related signaling pathways, regulation of the TME, and alteration of epigenetic mechanisms. These strategies not only inhibit the transition to a mesenchymal phenotype but also to suppress tumor proliferation and aggressiveness.

### 5.1 Pharmacological inhibition of EMT-associated signaling pathways

TGF-β/Smad and Wnt/β-catenin are integral to tumor cell proliferation, metastasis, and invasion (Hao et al., 2019; Xue et al., 2024; Springer Nature, 2026c). DKK family members (DKK1, DKK2 and DKK3) exhibit the ability to block Wnt signaling and inhibit cell growth and metastasis (Hirata et al., 2011; American Association for Cancer Research, 2026; Ueno et al., 2011). This inhibition occurs through promotion of β-catenin degradation and induction of apoptosis through non-canonical Wnt signaling (Hirata et al., 2011; American Association for Cancer Research, 2026; Ueno et al., 2011). In addition, Wnt inhibitors, such as ethyl acetate (EA), ciclopirox (CIC), and pyrrolidone (PO), have demonstrated strong anti-proliferative effects by inhibiting β-catenin activation in human RCC cell line models (Von Schulz-Hausmann et al., 2014). Excessive TGF-β/Smad activation is instrumental in initiating EMT (Sitaram et al., 2016). Small-molecule inhibitors targeting the TGF-β type I receptor, such as LY2109761 and LY2157299 (Galunisertib), have shown anti-metastatic activity and suppression of mesenchymal-like phenotypes across multiple cancer types, primarily in preclinical models (Melisi et al., 2008; Herbertz et al., 2015). These findings provide a promising direction for the treatment of targeting EMT in RCC with the potential to reverse therapeutic resistance.

### 5.2 Epigenetic reprogramming to reverse the EMT phenotype

Epigenetic dysregulation, including histone modification (histone deacetylase HDAC) and DNA methylation have a pivotal role in facilitating the EMT and in enhancing sunitinib resistance (Herbertz et al., 2015; Springer Natured; Bennett et al., 2011; Springer Nature, 2026e; Springer Nature, 2026f; He S. et al., 2022). Consequently, HDAC and DNA methylation inhibitors have garnered significant interest within the research community. In human RCC cell line models, HDAC leads to silencing of the tumor suppressor, *ASPP2*, which has been shown to reverse EMT by facilitating the formation of the β-catenin and E-cadherin triple complex at cell junctions (Van Hook et al., 2017; Park et al., 2010). The HDAC inhibitor, trichostatin A (TSA), significantly suppresses EMT and reduces invasion and migration (Wang et al., 2020). In RCC cell-line models, TSA has been reported to enhance the sensitivity of RCC to sunitinib through metabolic reprogramming (Sato et al., 2019). In addition, combination of the DNA methylation inhibitor, 5-aza-2′-deoxycytidine, with valproic acid exhibits a synergistic effect in inhibiting tumor proliferation and migration in human ccRCC cell lines by reversing EMT-related gene silencing (Xi et al., 2018). These findings highlight the therapeutic potential of HDAC and DNA methylation inhibitors, not only in counteracting EMT-related phenotypes, but in restoring drug sensitivity, thereby providing a promising strategy the treatment of RCC.

### 5.3 TME remodeling strategies to suppress EMT

The TME constitutes a dynamic network that profoundly influences EMT activation and therapy resistance (Aiello and Kang, 2019). TAMs and CAFs have pivotal roles in maintaining the mesenchymal phenotype through the release of cytokines, as well as remodelling of ECM (Aiello and Kang, 2019; Liu et al., 2024). These stromal cells not only facilitate EMT but also contribute to immune evasion, angiogenesis, and drug resistance (Wang et al., 2024). Consequently, targeting TAMs and CAFs represents a promising strategy to disrupt the microenvironment that supports EMT and to enhance the efficacy of therapeutic interventions. Recent research has demonstrated the development of novel therapeutic agents targeting TAMs. *In vitro* and *in vivo* preclinical studies in sarcoma have shown that the CSF1R inhibitor, pexidartinib (PLX3397), has been shown to enhance T cell infiltration and improve the anti-tumor immune response through reprogramming TAMs from a pro-tumoral M2 phenotype to a more immunostimulatory M1 phenotype, thereby indirectly restoring drug responsiveness (National Library of Medicine, 2026). Angiotensin receptor blockers (ARBs) have been shown to reprogram CAFs from an active state to a quiescent state by decreasing TGF-β with a hypoxia signal, subsequently reversing EMT and drug resistance in preclinical mouse tumor models (Fiori et al., 2019; Chauhan et al., 2019). Taken together, these approaches illustrate the potential of TME targeting components, providing novel avenues for clinical therapy.

### 5.4 Clinical strategies and future perspectives

While targeting EMT offers a promising avenue to overcome sunitinib resistance, prioritizing clinically feasible strategies over experimental concepts is crucial. Despite major advances, critical challenges in EMT persist. First, EMT should not be conceptualized as a binary switch but rather as a dynamic and continuous spectrum, which complicates detection and targeted therapeutic intervention. Second, although multiple biomarkers have been identified, there remains a scarcity of reliable indicators capable of effectively differentiating treatment responses among patients, thereby limiting the clinical utility of EMT as a prognostic tool. Lastly, the dynamic crosstalk between EMT and TME poses challenges in achieving sustained efficacy with signal-agent therapy. Future research should be focus on the following: (1) advancing real-time monitoring of EMT utilizing microfluidic platforms for the detection of CTCs; (2) developing clinically prognostic EMT biomarkers through mechanistic investigations of various signaling pathways; (3) and exploring tumor heterogeneity and the TME utilizing single-cell sequencing methodologies. Ultimately, as our comprehension of the EMT process continues to evolve, incorporation into clinical treatment strategies has the potential to significantly alter the therapeutic landscape for advanced RCC. Although these mechanisms have primarily been elucidated in preclinical models, emerging clinical trials indicate that EMT-associated characteristics, such as EMT/stroma/TGF-β programs and sarcomatoid phenotypes, influence sunitinib efficacy, the trajectory of resistance, and toxicity profiles. We summarize representative clinical studies reported to date, outlining eligibility criteria, treatment regimens, key outcomes, EMT-related readouts, and adverse events to facilitate a systematic appraisal of these relationships from a clinical-evidence perspective (Table 2).

**TABLE 2:** Studies linking EMT/Aggressive features with sunitinib response in RCC.

Enrollment criteria	Sunitinib regimen	Key outcomes	EMT/Invasive features	Adverse events	Ref.
72 patients with primary RCC (some metastatic) eligible for partial nephrectomy were given neoadjuvant sunitinib	Sunitinib 50 mg daily in 6-week cycles (4 weeks on, 2 off)	32% median reduction in primary tumor area. Lymph node metastasis was associated with a reduced tumor response rate	Presence of LN metastases, high grade, and non-clear-cell histology was associated with poorer tumor response	No unexpected toxicity, and surgery was not delayed due to sunitinib. Grade ≥3 surgical complications in 7%	Lane et al. (2015)
23 patients with advanced/metastatic RCC containing sarcomatoid dedifferentiation were treated with first-line sunitinib	Standard first-line TKI schedule (Sunitinib 50 mg qd,4/2)	Median PFS was 5.7 months and OS was 15.7 months. Objective response rate (ORR) was 30%. The percentage of sarcomatoid component did not significantly impact outcome	Sarcomatoid differentiation was examined. Performance status was a stronger determinant of response than sarcomatoid extent	No unique adverse-event trends noted beyond typical TKI toxicity	Kunene et al. (2014)
77 patients with metastatic RCC were treated with first-line sunitinib	Sunitinib was given continuously daily	Median PFS was 13 months and OS was 25 months. Metastatic RCC with sarcomatoid differentiation was associated with poorer OS and PFS, as well as unfavorable outcomes following chemotherapy and immunotherapy	Sarcomatoid features on pathology were specifically associated with inferior outcomes on sunitinib	Not reported in detail	Yildiz et al. (2013)
Including 87 patients with metastatic RCC containing sarcomatoid features. Based on histological subtype, the proportion of sarcomatoid differentiation, and performance status, patients were stratified into three risk groups	Arm A: Gemcitabine 1,000 mg/m^2^ IV (D1, 8, 22, 29), sunitinib 37.5 mg PO qd (D1–14, 22–35), q42d × up to 1 year Arm B: Sunitinib 50 mg PO qd (D1-14, 22–35), q42d × up to 1 year	Arm A: Median PFS was 4.5 months, and OS was 9.4 months Arm B: Median PFS was 3.6 months, and OS was 7.8 months	Sarcomatoid differentiation was a central inclusion criterion and used for risk stratification. Sarcomatoid histology was confirmed as an aggressive phenotype with generally poor outcomes	Worst grade (3/4/5):Arm A 25/10/1 and Arm B 16/1/0 The addition of gemcitabine increased cytopenias and other chemo-related toxicities	Carthon et al. (2023)
100 metastatic ccRCC patients were treated with first-line sunitinib (divided into a discovery set of 53 and validation of 47)	Standard first-line TKI schedule (Sunitinib 50 mg qd, 4/2)	Patients were stratified into four molecular subtypes (ccRCC1-4). Among them, the ccRCC4 subtype (characterized by a sarcomatoid-like phenotype and a more inflamed state) exhibit significantly shorter PFS and OS, as well as a lower ORR compared with ccRCC2 and ccRCC3	The poorest-prognosis group exhibit a sarcomatoid-like phenotype with greater invasiveness and showed intrinsic resistance to sunitinib, whereas tumors retaining more epithelial features respond better	Not reported in detail	Clinical Cancer Research (2026b)
55 patients with metastatic RCC were treated with first-line sunitinib, and tumor samples were analyzed retrospectively	First-line sunitinib, regimen not reported	CD44 was associated with sunitinib resistance, and CD44 positivity correlated with shorter PFS and increased incidence of sarcomatoid change	CD44 was associated with EMT-related transcription and sarcomatoid changes, which might explain the reduced sensitivity to sunitinib observed in CD44-driven tumors	Not reported in detail	Sekino et al. (2021)
80 patients with metastatic clear-cell RCC were treated with first-line sunitinib. Tumor tissues were assessed for Ezrin expression by IHC.	Standard first-line TKI schedule (Sunitinib 50 mg qd,4/2)	High Ezrin expression was significantly associated with aggressive disease and worse outcomes. Median OS in patients with Ezrin overexpression was only 12 months, vs. 27 months in low-Ezrin patients. High Ezrin correlated with more rapid progression, and patients who progressed within 3 months had markedly higher Ezrin levels than those with later progression	Elevated Ezrin expression in tumors signified a highly invasive, mesenchymal phenotype and was linked to poor response to sunitinib. This suggested EMT-related cytoskeletal changes (via Ezrin) contributed to sunitinib resistance in ccRCC.	Not reported in detail	Cetin et al. (2021)

## 6 Discussion and conclusion

EMT represents a fundamental mechanism underlying the development acquired sunitinib resistance in RCC. During this process, tumor cells undergo a transformation from an epithelial phenotype to a more aggressive mesenchymal phenotype, enhancing the ability to evade the effects of the drug and promote survival. EMT-associated reprogramming confers RCC with a multifaceted resistance to sunitinib. Specifically, EMT activates pro-survival signaling and enhances the expression of ATP-binding cassette transporters, facilitating the efflux of sunitinib from cells. This process induces metabolic reprogramming and angiogenesis, thereby protecting tumor cells from oxidative stress. In response to immune cell attacks, EMT increases the expression of PD-1/PD-L1 on the tumor surface and upregulates anti-apoptosis proteins, thereby creating a protective microenvironment. Furthermore, by inducing a stem-like state, EMT facilitates the transformation of tumor cells into cancer stem cells that are characterized by significant therapeutic resistance and tumor-initiating capabilities, ultimately contributing to tumor relapse, even following sunitinib treatment.

Addressing EMT-associated resistance presents a formidable challenge and offers avenues for the development of innovative therapeutic strategies. Investigation of EMT-targeted therapies has garnered considerable attention, which has the potential to restore drug sensitivity and improve patient outcomes through inhibition of the EMT process. Within this framework, three major approaches have emerged as promising therapeutic options: targeting EMT-related signaling pathways, modulating the TME, and employing epigenetic reprogramming. These strategies aim to effectively suppress the EMT and provide opportunities to enhance the therapeutic efficacy of sunitinib in RCC. In summary, EMT contributes to the development of acquired sunitinib resistance in RCC by enhancing tumor adaptability and survival. Targeting the EMT to counteract sunitinib resistance presents a promising avenue. Ongoing research is essential for translating these findings into clinical applications. By simultaneously addressing the multiple characteristics of EMT, future strategies may help prevent resistance and improve long-term outcomes for patients with advanced RCC.
